# Aboriginal Australian mitochondrial genome variation – an increased understanding of population antiquity and diversity

**DOI:** 10.1038/srep43041

**Published:** 2017-03-13

**Authors:** Nano Nagle, Mannis van Oven, Stephen Wilcox, Sheila van Holst Pellekaan, Chris Tyler-Smith, Yali Xue, Kaye N. Ballantyne, Leah Wilcox, Luka Papac, Karen Cooke, Roland A. H. van Oorschot, Peter McAllister, Lesley Williams, Manfred Kayser, R. John Mitchell, The Genographic Consortium, Syama Adhikarla, Christina J. Adler, Elena Balanovska, Oleg Balanovsky, Jaume Bertranpetit, Andrew C. Clarke, David Comas, Alan Cooper, Clio S. I. Der Sarkissian, Matthew C. Dulik, Jill B. Gaieski, ArunKumar GaneshPrasad, Wolfgang Haak, Marc Haber, Angela Hobbs, Asif Javed, Li Jin, Matthew E. Kaplan, Shilin Li, Begoña Martínez-Cruz, Elizabeth A. Matisoo-Smith, Marta Melé, Nirav C. Merchant, Amanda C. Owings, Laxmi Parida, Ramasamy Pitchappan, Daniel E. Platt, Lluis Quintana-Murci, Colin Renfrew, Ajay K. Royyuru, Arun Varatharajan Santhakumari, Fabrício R. Santos, Theodore G. Schurr, Himla Soodyall, David F. Soria Hernanz, Pandikumar Swamikrishnan, Miguel G. Vilar, R. Spencer Wells, Pierre A. Zalloua, Janet S. Ziegle

**Affiliations:** 1 Department of Biochemistry and Genetics, La Trobe Institute for Molecular Sciences, La Trobe University, Melbourne, Victoria, Australia; 2 Department of Genetic Identification, Erasmus MC University Medical Center Rotterdam, The Netherlands; 3 Australian Genome Research Facility, Melbourne, Victoria, Australia; 4 Biotechnology and Biomolecular Sciences, University of New South Wales, New South Wales, Australia; 5 School of Biological Sciences, University of Sydney, Sydney, Australia; 6 The Wellcome Trust Sanger Institute, Welcome Trust Genome Campus, Hinxton, Cambridgeshire, United Kingdom; 7 Office of the Chief Forensic Scientist, Victoria Police Forensic Services Department, Melbourne, Victoria, Australia; 8 Griffith University, Queensland, Australia; 9 Community Elder and Cultural Advisor, Brisbane, Queensland, Australia; 10 Madurai Kamaraj University, Madurai, Tamil Nadu, India; 11 University of Adelaide, South Australia, Australia; 12 Research Centre for Medical Genetics, Russian Academy of Medical Sciences, Moscow, Russia; 13 Universitat Pompeu Fabra, Barcelona, Spain; 14 University of Otago, Dunedin, New Zealand; 15 Department of Anthropology, University of Pennsylvania, PA, USA; 16 Lebanese American University, Chouran, Beirut, Lebanon; 17 National Health Laboratory Service, Johannesburg, South Africa; 18 IBM, Yorktown Heights, NY, USA; 19 Fudan University, Shanghai, China; 20 University of Arizona, Tucson, AZ, USA; 21 Institut Pasteur, Paris, France; 22 University of Cambridge, Cambridge, United Kingdom; 23 Universidade Federal de Minas Gerais, Belo Horizonte, Minas Gerais, Brazil; 24 National Geographic Society, Washington, DC, USA; 25 IBM, Somers, NY, USA; 26 Applied Biosystems, Foster City, CA, USA

## Abstract

Aboriginal Australians represent one of the oldest continuous cultures outside Africa, with evidence indicating that their ancestors arrived in the ancient landmass of Sahul (present-day New Guinea and Australia) ~55 thousand years ago. Genetic studies, though limited, have demonstrated both the uniqueness and antiquity of Aboriginal Australian genomes. We have further resolved known Aboriginal Australian mitochondrial haplogroups and discovered novel indigenous lineages by sequencing the mitogenomes of 127 contemporary Aboriginal Australians. In particular, the more common haplogroups observed in our dataset included M42a, M42c, S, P5 and P12, followed by rarer haplogroups M15, M16, N13, O, P3, P6 and P8. We propose some major phylogenetic rearrangements, such as in haplogroup P where we delinked P4a and P4b and redefined them as P4 (New Guinean) and P11 (Australian), respectively. Haplogroup P2b was identified as a novel clade potentially restricted to Torres Strait Islanders. Nearly all Aboriginal Australian mitochondrial haplogroups detected appear to be ancient, with no evidence of later introgression during the Holocene. Our findings greatly increase knowledge about the geographic distribution and phylogenetic structure of mitochondrial lineages that have survived in contemporary descendants of Australia’s first settlers.

The human colonisation of Australia occurred relatively soon after the migration of anatomically modern humans out of Northeast Africa some 60 to 80 thousand years ago (KYA)^1^,^2^,^3^. This initial settlement of Australia occurred between 47–55 KYA, based on the dating of archaeological sites dispersed throughout the continent^3^,^4^,^5^,^6^,^7^, and the analysis of contemporary Aboriginal Australian DNA^8^,^9^,^10^,^11^,^12^,^13^,^14^,^15^,^16^,^17^,^18^.

Although there is consensus about the time the ancestors of Aboriginal people arrived in the ancient landmass of Sahul (which comprised Australia and New Guinea), there is debate over the route(s) taken by them to reach Sahul^9^,^10^,^11^,^18^,^19^,^20^. This is mainly because the genetic structure of present-day Aboriginal Australians and New Guineans is different, implying a long separation that started at least 30 KYA^9^,^15^,^21^. Did the colonisers enter Sahul via present-day New Guinea and subsequently spread southwards to Australia, or were there different routes into Sahul, such that one or more groups entered Australia via the ancient northwestern coast that is now submerged under the Timor and Arafura Seas^19^? Furthermore, a mix of genetic^22^,^23^,^24^, archeological^25^, anthropological^26^,^27^ and linguistic data^28^ have suggested later migration(s) to Australia in the Holocene epoch, particularly from the Asian sub-continent.

Although mainly based on small sample sizes and few sampling locations, DNA studies have revealed the distinctiveness of Aboriginal Australians. Mitochondrial DNA (mtDNA) lineages of all three major mtDNA clades found outside Africa (macrohaplogroups M, N and R- macrohaplogroup R lineages lie within N; see http://phylotree.org^29^) are present in Aboriginal Australians. Subsequent to their arrival in Australia, the M founder types diversified into the Australian-specific haplogroups present today, including M42a and M15 (and possibly M14), and the N founder types into N13, N14, O and S as reviewed in ref. 30, whilst the one lineage within macrohaplogroup R, haplogroup P, most probably evolved either in Sunda (the ancient landmass comprising present-day Island Southeast Asia) just prior to the colonisation of Sahul^31^,^32^,^33^ or in northern Sahul (New Guinea)^34^. Subsequently, P evolved into sublineages that are unique to New Guinea or to Australia, respectively.

What is apparent from the limited Aboriginal Australian mtDNA data currently available is that it lacks phylogenetic resolution and geographic coverage. Specifically, most studies of mtDNA diversity have focused on populations of northern Australia^9^,^21^,^23^,^35^,^36^,^37^,^38^. The exceptions are van Holst Pellekaan, *et al*.^39^ and Presser, *et al*.^40^, which analysed samples from New South Wales and Tasmania, respectively. A recent report on the genomic history of Aboriginal Australians^18^ included 83 mitogenomes drawn widely from the continent. However, while the mitochondrial haplogroup affiliation of the individuals is given, sequence data were not available.

A relatively large number of Aboriginal mtDNAs have also been reported as haplogroup M, N or P “unclassified” (also denoted as M*, N* or P*). This has occurred because either only the hypervariable control-region segments (HVS-I and HVS-II) were sequenced and/or the mtSNPs genotyped proved insufficient for further subhaplogroup assignment^8^,^9^,^17^,^35^,^36^,^41^. The paucity of knowledge is also illustrated by the fact that, to date, there are only 39 Aboriginal Australian mitogenomes in GenBank (0.16% of all human mitogenome sequences as of June, 2016).

The 127 newly described mitogenome sequences of the present study comprise part of the Genographic Project during which samples from Aboriginal Australians were collected for analysis of uniparental DNA variation^14^,^17^. Participants were drawn especially from those areas of Australia, either previously not sampled (Queensland and Victoria), or very poorly represented (Tasmania). These data were used to further clarify the phylogenies of Aboriginal Australian mitochondrial haplogroups, their distribution within the continent, and potentially make inferences regarding the colonisation and migration routes taken by the maternal ancestors of present-day Aboriginal Australians.

## Results and Discussion

The samples selected for mitogenome sequencing are a subset within a larger mtDNA sample set described in Nagle, *et al*.^17^. The haplogroup assignments of the present sample based on initial mtSNP genotyping are given in Supplementary Table 1. The frequencies of each haplogroup as well as geographical distributions are very similar to those observed in the much larger Aboriginal sample of mtDNAs reported in Nagle, *et al*.^17^ (Supplementary Table 2).

There is uncertainty as to the traditional homeland of many individuals in this study as a result of the great dislocation of Aboriginal society since European colonization started in 1788. This disruption included forced removal from their homelands into settlements, restrictions on marriage and forced removal of children. Accordingly, any attempt at the reconstruction of the historical genetic structure of Aboriginal Australia must be mindful of the past treatment over the last 200 years.

Detailed phylogenetic trees using full mitogenome sequences were constructed for the following haplogroups: a) M14, M15, M16, M42 and Q b) N13, N14, O and S and c) R12 and P (Supplementary Figures 1, 2, 3). In addition, a simplified tree showing the overall basic phylogeny and diagnostic mtSNPs of the major Australian sub-branches of macrohaplogroups M and N (including R) are shown in Fig. 2a and b, respectively. Haplogroup nomenclature follows that of PhyloTree Build 17 (van Oven and Kayser, 2009) and logically expands on this nomenclature for newly identified (sub)haplogroups.

### Phylogeny of lineages within macrohaplogroup M

**Figure 1:**
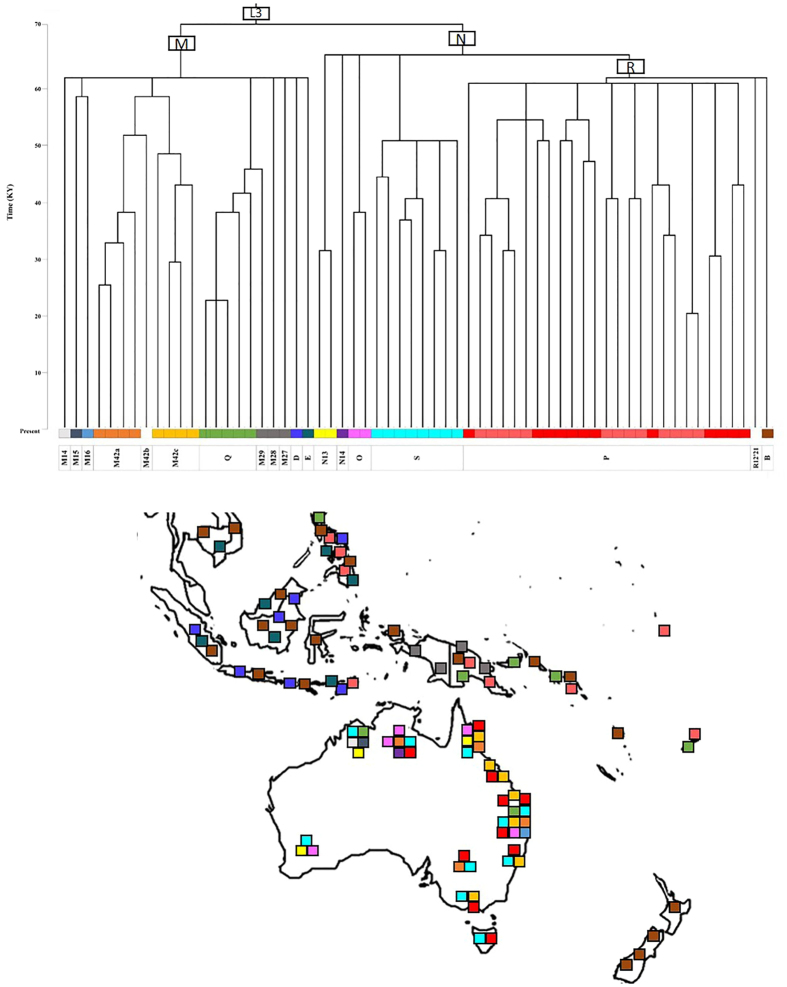
Phylogeny and geographical distributions of Aboriginal Australian mtDNA lineages and those of the surrounding regions of past and present studies. Length of branches indicative of time since divergence. Colored squares on map indicate presence of specific haplogroups depicted in phylogeny.

This tree comprised the 34 haplogroup M mitogenomes of this study and the published M14, M15, M42a, M42b, Q1, Q2 and Q3 mitogenomes^8^,^9^,^37^,^38^,^42^,^43^,^44^ (provisional diagnostic SNPs are presented in Supplementary Figure 1). Notably, construction of the tree revealed a novel subclade within M42 (here labelled M42c) that is, according to available data, unique to Aboriginal Australians. In addition to A9156G, which is a definer of haplogroup M42, M42c is defined by nucleotide positions (np) C64T and T195C, and the absence of G8251A (which is shared by M42b and some M42a mitogenomes). M42c has at least two subtypes, with M42c1 found in Queensland and M42c2 found in Queensland, New South Wales and Victoria (Fig. 1). It is unclear whether the haplogroup labelled ‘M42’ in Malaspinas, *et al*.^18^ is identical to M42c because of the lack of sequence data. Distinct subclades were also identified within the previously known haplogroup M42a, namely M42a1 which, in turn, is subdivided into M42a1a and M42a1b (Supplementary Figure 1).

The existence of a novel branch within the M42 clade was first suggested by Ballantyne, *et al*.^35^, in which some samples were labelled M42*(xM42a) because they carried the transition at np A9156G (the defining mutation of M42) but not G12771A (one of the defining mutations of M42a). Haplogroups M42a and M42c are, by far, the major representatives of haplogroup M in Australia and both appear to be widespread across the continent (Fig. 1).

Haplogroup M42a has previously provoked interest because its apparent sister clade, M42b, was found in tribal group(s) in southern India^42^,^43^ as well as in single individuals from Iran^45^, Saudi-Arabia^46^ and Mauritius^46^. Time-to-most recent-common-ancestor (TMRCA) estimates of M42a and M42b suggest that they diverged some 50 KYA, possibly in India^42^, with the ancestors of Aboriginal Australians continuing southeastward to Sahul^47^,^48^. However, M42a and M42b are only tenuously linked as they share just two mutations, 9156G and 8251A, with the latter being a well-known mutational hotspot with 26 independent occurrences in PhyloTree Build 17^29^. Indeed, two of the published M42a mitogenomes lack the 8251 transition, suggesting back mutations have occurred at this position^8^,^42^,^49^. Hence, the sharing of 8251A between M42b and some of the M42a lineages might be coincidental (identical-by-state) rather than indicative of phylogenetic relatedness (identical-by-descent). In contrast, the A9156G transition appears is mildly recurrent, with only five occurrences in the entire PhyloTree Build 17. Thus, M42a, and M42c are more likely to be identical-by-descent.

Other indigenous M subhaplogroups in Aboriginal Australians, M15 and possibly M14^9^, appear to be much less frequent than haplogroups M42a and M42c as we did not find haplogroup M14 in our dataset and haplogroup M15 was only observed once. This additional M15 sequence, from Western Australia, together with the only other published haplogroup M15 sequence^9^, helped to refine the diagnostic motif of this haplogroup to nine mutations (Supplementary Figure 1). One individual from Queensland had a novel M mitogenome that shared individual mutations, with existing haplogroups (i.e., M29, M52, M82, M28, M80, M53, M15, M23, M50), but did not show a convincing phylogenetic link to them.

**Figure 2:**
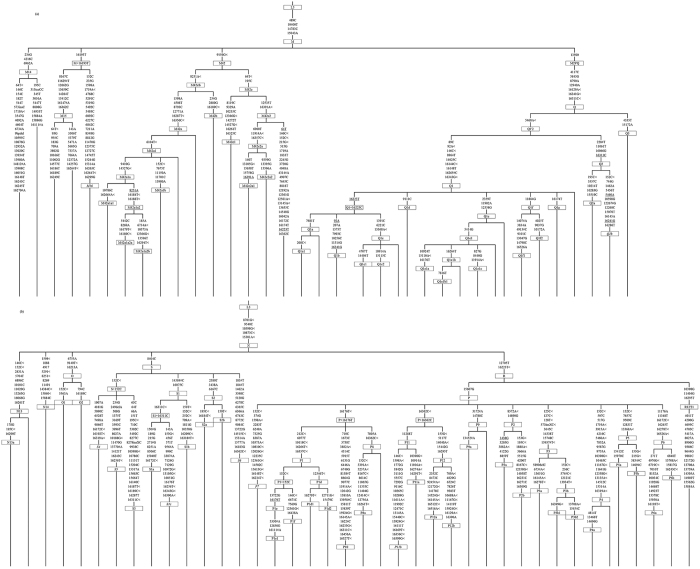
Schematic tree of (**a**) macrohaplogroup M and (**b**) macrohaplogroup N lineages including those specific to Aboriginal Australians. Diagnostic control-region and coding region positions are as indicated.

Interestingly, this novel mitogenome shared variant C16193T with haplogroup M15 but there was uncertainty as to whether they shared it by descent and, therefore, this mitogenome was tentatively labelled as haplogroup M16 (Fig. 2a).

Haplogroup Q is of interest because of its estimated age of at least ~37 KY^50^ and its relatively high frequency in Australia’s nearest neighbours, New Guineans, Timorese and Island Melanesians^21^,^34^,^51^,^52^. This haplogroup has three known subclades; Q1, Q2 and Q3, all of which are found in New Guineans/Island Melanesians, with Q1 and Q3 being additionally present in Timorese^34^. To date, haplogroup Q has been found only in a single Aboriginal Australian person and it was a unique variant within haplogroup Q2, labelled Q2b^9^.

The three haplogroup-Q individuals detected in the current study all belonged to Q1. One of them belonged to a newly proposed subhaplogroup, Q1g, shared with an individual from the Solomon Islands^53^ and the other two belonging to haplogroup Q1a (Supplementary Figure 1). While all of them were sampled in Far North Queensland, the three Q individuals were aware they had Torres Strait Island maternal ancestry. Some of the Torres Strait Islands (which lie between Australia and New Guinea) are an integral part of the nation of Australia, with the remainder belonging to Papua New Guinea. Although Aboriginal Australians are culturally and linguistically distinct from Torres Strait Islanders, there are reports of trading and intermarriage^54^. Any such gene flow, however, was not detected in our small sample of mitogenomes.

### Phylogenies of lineages within macrohaplogroup N

Haplogroups N13 and O show the most geographically restricted distribution of the major observed haplogroups in Australia. Haplogroup N13, however, has a wider distribution than previously thought, with a presence in Queensland as well as Western Australia. One Queensland individual’s mitogenome was similar to a sequence from Western Australia^11^, whilst the other individual was more divergent from other N13 mitogenomes. The increased number of haplogroup-N13 mitogenomes has allowed the identification of a novel subclade, N13a, which in turn has a subclade N13a1 (Fig. 2b).

Although haplogroup O has a wide distribution within Australia, being present in Queensland, Western Australia and the Northern Territory, it was not observed in high frequency compared to other widespread Australian haplogroups (Fig. 1). The increased number of haplogroup-O mitogenomes resulted in the identification of a novel subclade within haplogroup O1a (i.e., O1a1) and the newly defined haplogroup O2. Haplogroup O1a was found in all three northern States, while haplogroup O2 was present in the Northern Territory and Queensland (Supplementary Figure 2).

Haplogroup S evolved within Australia and has five recognized subtypes, S1 to S5; although, of these, S4 and S5 are each represented by only one mitogenome sequence^9^,^37^. However, haplogroup S5 has been reported in Western Australia^18^. Our additional haplogroup-S mitogenomes belonged to S1a, to the newly proposed S1b, to the tentatively defined S1c and to S2 (Fig. 2b). S1 had a wide distribution, being present in Queensland, New South Wales, Western Australia and the Northern Territory. The single S1c subtype was from Queensland, and its provisional diagnostic SNPs are provided in Supplementary Figure 2. S2 also had a wide distribution, being found in all States, including the island of Tasmania (Fig. 3). Novel subclades within haplogroup S2 were identified, and were labelled S2a and S2b (Supplementary Figure 2). Haplogroups S3, S4 and S5 were not observed in our sample. However, we did detect a novel haplogroup S mitogenome (from New South Wales) which we provisionally denoted S6 (Fig. 2b).

### Phylogeny of lineages within macrohaplogroup R

Haplogroup P is an ancient haplogroup with ten recognized subclades thus far (P1 to P10) (Fig. 2b). The haplogroup is found in Island Southeast Asia (ISEA), in particular the Philippines, and also in New Guinea, Island Melanesia and Australia^9^,^18^,^31^,^32^,^34^,^37^,^38^,^51^,^55^.

Although the known two subclades of haplogroup P3 were thought to have separate geographical distributions, with P3a restricted to Australia and the majority of P3b individuals (2 out of 3) to New Guinea^9^,^37^,^51^, our results revealed three P3b individuals, who resided in Queensland or New South Wales, but have known Torres Strait Islander maternal ancestry (Supplementary Figure 3).

The subclades of haplogroup P4 have also been thought to show geographical differences, with P4a restricted to New Guinea and P4b to Australia^8^,^9^,^37^,^51^. There was no evidence of P4a in our Aboriginal Australian sample. However, after sequencing five haplogroup P4b individuals it was apparent that P4a and P4b shared too few mutations for both subtypes to remain within the same clade, P4, and that P4b was more parsimoniously joined (through mutation C11288T) with a single haplogroup P lineage from Tasmania, together forming a newly proposed haplogroup, which we designated P11. As a result of this rearrangement, those individuals that were previously assigned to P4b (Aboriginal Australians) have been allocated to the subclade P11 to reflect its uniqueness. Haplogroup P11 has a wide distribution in Australia, with representative mitogenomes from all States where data were available (Fig. 1). The eight mitogenomes of P11 can be further classified into subtypes; P11a and P11b, with P11a found on mainland Australia and P11b represented in a single individual from Tasmania. This new haplogroup assignment also receives support from maximum likelihood analysis (Supplementary Figure 4). As a result of this analysis, the New Guinean-specific haplogroup P4a now becomes P4.

In our sample, there were individuals who carried haplogroup P1e, which was previously found in Timor and New Guinea^34^,^38^, and New Guinean-specific haplogroup P2^36^,^37^,^44^,^51^,^55^. Notably, these individuals traced their maternal ancestry to the Torres Strait Islands, and may therefore have (ultimate) maternal ties to the New Guinea mainland. The New Guinean and Torres Strait Islander haplogroup-P1e mitogenomes shared three mutations not present in the Timorese P1e lineage and, therefore, the former has been denoted as a novel clade, P1e1. Interestingly, within haplogroup P2, our new sequences distinguished two subclades, P2a and P2b, with P2a restricted to New Guinea and P2b to the Torres Strait Islanders (Supplementary Figure 3).

This study greatly improved the substructure of haplogroup P5 (previously merely represented by a single sequence from the Northern Territory^51^), with two major subclades which are predominantly found in Queensland: P5a and P5b; the first of which appeared to be more common (Supplementary Figure 3). Four haplogroup-P6 mitogenomes from Victoria and Queensland indicated that this haplogroup is not restricted to the Northern Territory^37^. Our increased sampling further resolved the haplogroup P6 phylogeny, with two subclades (P6a and P6b) evident. There was no evidence of haplogroup P7 in our sample, previously reported in the Northern Territory^37^. The sequencing of an additional haplogroup P8 mitogenome, together with sequence AY289055 previously thought to belong to haplogroup P6, allowed us to propose a basal haplogroup-P8 motif, as well as the recognition of a subtype P8a, and a TMRCA estimate of this haplogroup.

The remaining 13 haplogroup-P mitogenomes belonged to a novel clade, which we denoted P12 (Fig. 2b), with all these individuals residing in Queensland. Two subhaplogroups, P12a and P12b, could also be identified amongst the sequences (Supplementary Figure 3).

In our sample we did not detect haplogroup R12, previously observed in a single individual from the Northern Territory^44^ and recently in three individuals in Western Australia^18^.

### TMRCA estimates

The TMRCA estimates of the Aboriginal Australian haplogroups based on two different mutation rates are shown in Table 1. The mutation rate of Soares, *et al*.^56^ reflects an estimated ‘evolutionary’ mutation rate, whereas that of Fu, *et al*.^57^ is calibrated with ancient-DNA samples. The present study’s estimates differed from those calculated by Behar, *et al*.^50^, Hudjashov, *et al*.^9^ and van Holst Pellekaan^30^ due to a difference in methodologies and increased sampling from different regions. Several estimates have large confidence intervals due to increased variability of the mitogenomes as well as low numbers.

The estimates generated by the two methods were similar for most haplogroups, especially for those represented by more than four mitogenomes. Haplogroups represented by less than four mitogenomes showed larger differences. Overall, TMRCA estimates using Soares, *et al*.^56^ mutation rate were older than those generated using that of Fu, *et al*.^57^. The TMRCA estimates of all the uniquely Aboriginal haplogroups are consistent with the conclusion of the recent review of archaeological evidence that the initial colonisation of Sahul occurred at least 47 KYA^3^ with the age estimates using Fu, *et al*.^57^ fitting well with this date. The Fu, *et al*.^57^ mutation rate may be considered the most accurate since it was calibrated using reliably dated ancient DNA samples taken from modern human remains whose ages span the last 40 KY, and come from different locations throughout the world. Colonisation even earlier than these dates, however, cannot be completely rejected since the large expansion of land formerly comprising the north western part of Australia has been submerged since ~8 KYA^58^ and this region may well have been colonised earlier than 47 KYA. In addition, there is an inherent difficulty in finding archaeological evidence of human occupation of Australia before 55 KYA due to the continent’s harsh arid environment^3^,^7^,^59^,^60^.

The TMRCA for haplogroup S is between 49 and 51 KYA and it may have been one of the first Australian-specific haplogroups to diversify after it evolved from its ancestral N type, which is consistent with the fact that the root of S is only one mutation step from N. The recent mitogenome sequencing of the skeletal remains of WLH4 of Lake Mungo, New South Wales^16^ has determined that this individual (chronologically dated to the late Holocene by Durband, *et al*.^61^) belonged to haplogroup S2. Our analysis suggests that she belongs to subtype S2a1a, with her closest three maternal living descendants also living in New South Wales, with one individual, W26, residing close to Lake Mungo.

The common ancestor of the M42a and M42c evolved 50–53 KYA (Table 1) and this date may serve as an upper bound to the date of colonization of Australia. The novel haplogroup M42c is 5–10 KY older than M42a.

Haplogroup P is the oldest and most polytomous of the Australian haplogroups, with a TMRCA estimate of 60 KY (Table 1). In the newly reconstructed P phylogeny comprising 112 mitogenomes, we find that the Australian-specific haplogroups are as ancient as those found in New Guinea and Philippines, but all estimates have large confidence intervals. The ‘novel’ P subtypes identified in this study P11 (including the former P4b) is 50 KY old and P12 is 46 KY old.

The antiquity of Australian P lineages makes identifying the geographic origin of P more problematic and, perhaps, these findings strengthen the hypothesis it evolved within Sahul. Importantly, given that some of the Australian-specific P lineages (P8, P11 and P12) are at least 45 KY old, these data support a long separation of Australian and New Guinean populations. This deep divergence between New Guineans and Aboriginal Australians is also supported by Y-chromosome data suggesting that the two populations may have separated at least 48 KY ago^14^,^15^.

Recently, it has been argued, based on genome wide analysis of samples from mainly northern and western Australia, that first settlement of Australia occurred less than 42 KYA^18^. However, the authors acknowledged that this date was not congruent with current archeological evidence. Our genetic data suggest a much earlier arrival of the ancestors (no later than 45 KYA) as does the recent Y-chromosome study^15^. Further, very recent archeological evidence supports occupation of the interior of Australia at least 49 KYA^60^.

### Migrations

While it is presently unknown where P evolved in this region, it is likely that haplogroup P carrying females entered Australia from present-day New Guinea, and this haplogroup rapidly diverged into sub haplogroups. Today, Australia contains a number of unique P subtypes (P5, P6, P7, P8, P11 and P12), while P2 is distantly shared between New Guinea and the Torres Strait Islands and P3 appears in mainland Australia, Torres Strait Islands and New Guinea.

If we assume that haplogroup M42b belongs to a separate clade to that comprising M42a’c, then the ancestor of the latter most probably evolved in the Near East or South Asia and reached Sahul via the southern route into Sunda^47^,^48^. Subsequently, the ancestors moved through the string of Indonesian islands, including Nusa Tenggara and Timor. It is possible that this M42a’c ancestor arrived in Sahul at least 50 KYA, via the coastline of northwest Australia that is now submerged beneath the Timor and Arafura Seas, rather than via New Guinea. Once in the continent, their descendants subsequently diverged into M42a and M42c. Importantly, the female descendants of M42a’c may never have spread to New Guinea, as the M42 clade has not thus far been detected in present-day New Guineans^23^,^62^,^63^.

It is also plausible that the N* ancestors of equally ancient Aboriginal maternal lineages such as N13, S and O may have followed a similar route of entry to that of the ancestors of M42a and M42c, as these lineages are also not shared, as far as we know, with modern New Guineans^23^,^37^,^62^. Whether or not females carrying ancestral N lineages followed a similar route(s) to that taken by the female M ancestors is unknown, but, clearly the descendant haplogroups of both M* and N* are distinct from those observed among modern New Guineans.

It has been argued that the M42a’c and N* ancestral females may have taken a more northerly route (like that proposed for the Australian haplogroup P ancestors) through Sunda into Sahul via present day New Guinea, and later their descendants extended into Australia, where the M42a, M42c, N13, O, S and Australian-specfic P subtypes evolved *in situ*^9^,^30^. Alternatively, those mitochondrial haplogroups presently found only in Aboriginal Australians may well have been present in ancient New Guineans but have been lost through genetic drift.

There is also considerable debate over whether Australia received migrants from other populations, particularly from South Asia, during the mid-Holocene. The postulated Indian connection arises from some archaeological^25^ and linguistic findings^28^,^64^, as well as DNA analyses^22^,^23^,^24^. In particular, immigrants have been postulated as necessary to explain the introduction of the dingo (Australian dog) ~5 KYA, and the microlith tradition^25^,^27^. Claims of linguistic similarities between Dravidian languages of southern India and the Pama-Nyungan language of most of Aboriginal Australia have also been used to suggest a connection between the two regions^28^. However, the hypothesized connection to India has been stongly disputed^65^.

Redd and Stoneking^23^ had previously suggested mtDNA lineages of Aboriginal Australians were most closely related to those in southern India, and later, Redd, *et al*.^22^ claimed that the Y-chromosome haplogroup C-RPS4YT indicated a Holocene connection between the males of Southern India/Sri Lanka and Australia, although both these studies have relatively low data resolution. More recently, using a genome-wide analysis, Pugach, *et al*.^24^, calculated that a considerable component (~11%) of the Aboriginal Australian genome could be attributed to a South Indian migration to Australia some 4 KYA. By contrast, all studies on mtDNA variation in Aboriginal Australians, including the present one, find no evidence of recent gene flow from the Indian sub-continent during the Holocene. In fact, the mitogenomes of the current study show no evidence for sharing of Aboriginal haplogroups with those found in the Asian sub-continent except for haplogroup M42, although the TMRCA estimates for M42a and M42c (Australia) and M42b (South Asia) suggest these clades split long before the Holocene.

Our findings concur with those of other studies, that there is no evidence of back migration of females into New Guinea after novel haplogroups arose in Australia^23^,^30^,^37^,^63^,^66^. However, what adds to the complexity of any reconstruction of the past is the effects of the Austronesian expansion from (probably) Taiwan through Island Southeast Asia, then via New Guinea into Oceania, and the Pacific some 4 KYA. However, a recent study^67^ confirms that this expansion was a rapid coastal migration resulting in little admixture with the indigenous populations of New Guinea.

The traditional Aboriginal marriage pattern of patrilocality and the movement of women along ‘Songline’ routes over large distances^68^ for hundreds, if not thousands of generations can explain the wide geographical spread of indigenous haplogroups M42a, M42c, S, N13, O and P subtypes. The explanation for some very low-frequency ancient mitochondrial lineages in Aboriginal Australians remains speculative, but possibly involves factors such as marked isolation and limited resource access of some communities over thousands of years and the consequently marked effects of random genetic drift in small bands. However, it is possible that these low frequencies are due to sample bias and that their frequencies may be higher elsewhere in regions not yet sampled.

As all Aboriginal-specific mitochondrial haplogroups are of great antiquity, show considerable substructure, and are (mostly) very widely dispersed across the Australian continent while not being present outside Australia, it can be inferred that after initial colonisation some 50 KYA there has been a very long period of isolation of humans in Australia.

## Subjects and Methods

### Sample collection

Saliva samples were collected using Oragene^®^ DNA Collection kits (DNA Genotek Inc. Ontario, Canada) from 127 self-declared Aboriginal Australians who had volunteered to participate in the “Genographic Project”. Informed consent was obtained by all participants of the “Genographic Project”. Further information on sample characteristics and treatment is given in Supplementary Document. Individuals were previously genotyped for selected mtSNPs to assign them to the major mitochondrial haplogroups^17^. Individuals resided in the States of Queensland (n = 103), New South Wales (n = 14), Victoria (n = 6), Tasmania (n = 2) and Western Australia (n = 2), with the majority of participants from the Eastern States of Australia (Supplementary Table 2). Although the majority of samples were collected in Queensland, a number of individuals indicated that their maternal ancestry lay in other regions or States. In particular, participants had maternal ties to Torres Strait Islands, Tasmania and New South Wales. It is important to note that Aboriginal Australian affiliation is culturally based, and not defined by a person’s genetic composition. This study was approved by the Human Ethics Committee of La Trobe University. All methods were carried out in accordance with the relevant guidelines and regulations. All experimental protocols were approved by the Human Ethics Committee of La Trobe University.

### Whole mitogenome sequencing

An input of 100 ng of genomic DNA extracted from Oragene^®^ collection kits (as per the manufacturer’s recommendations) was prepared and indexed for Illumina sequencing using the TruSeq DNA Sample Prep Kit (Illumina) as per manufacturer’s instruction. The library was quantified using the Agilent TapeStation and the Qubit™ RNA assay kit for Qubit 2.0^®^ Fluorometer (Life Technologies). The indexed libraries were then enriched for mtDNA and prepared for paired-end sequencing on a HiSeq instrument using the v3 100 cycle kit (Illumina) as per manufacturer’s instructions. For more detailed descriptions of the mitogenome data processing, genotyping and filtering, see the Supplemental Experimental Procedures.

### Data Analysis

The 127 mitogenomes were aligned to the revised Cambridge Reference Sequence (rCRS)^69^,^70^. Two different mutation rates were utilized to calculate TMRCA estimates. The mutation rate of Soares, *et al*.^56^ is based on the analysis of over 2,000 modern human mitogenomes whereas that of Fu, *et al*.^57^ is based on the analysis of ten mitogenomes of skeletal remains of ancient Eurasians spanning the last 40 KY. These mutation rates are based on the recalibration of the molecular clock of modern human and ancient human mitochondrial genomes, respectively.

Phylogenies of the haplogroups were constructed using the Network 5.0 software with the reduced median algorithm^71^. TMRCA estimates of haplogroups were also calculated using the rho (ρ) statistic^72^ based on phylogenetic rate of one mutation every 3624 years^56^. The mtDNA clock provided by Soares, *et al*.^56^ was subsequently used to correct for purification selection.

Bayesian inferences of the TMRCAs of haplogroups were calculated using BEAST^73^. The software jModeltest2^74^ was used to identify the best-fitting nucleotide substitution model, which was GTR+I+G. For each inference, three independent MCMC runs of 20,000,000 iterations each were performed and combined using LogCombiner v1.8.2 (included in BEAST package). We used one prior distribution for complete mitochondrial genomes incorporating Fu, *et al*.^57^ priors, 2.67 × 10^−8^ (2.16–3.16 × 10^−8^, 95% HPD). TRACER^75^ was employed to estimate the TMRCAs, to check convergence of the two BEAST runs of 60 million iterations and to compute the effective sample size (ESS) and the 95% confidence intervals for all parameters.

The 127 mitogenome sequences, together with all previously published mitogenome sequences from relevant haplogroups, were analyzed in a phylogenetic context by drawing maximum-parsimony trees using PhyloTree Build 17 (van Oven and Kayser, 2009) as reference. This allowed several phylogenetic improvements to be made and novel (sub)haplogroups identified in the process were given new haplogroup labels. Phylogenetic trees were constructed with the program mtPhyl (http://eltsov.org) and PhyloTree (Build 17)^29^. Thirteen of the 127 mitogenomes have also been subject to whole-genome sequencing at the Sanger Institute, using the protocols as described in Bergstrom, *et al*.^15^. These data confirmed the haplogroup assignments of these samples. Aboriginal Australian mitogenome sequences in GenBank that were included in the analysis were from Ingman, *et al*.^38^, Ingman and Gyllensten^37^, Kivisild, *et al*.^44^, van Holst Pellekaan, *et al*.^8^, Friedlaender, *et al*.^51^, Hudjashov, *et al*.^9^, Rasmussen, *et al*.^11^ and Heupink, *et al*.^16^. The sequence data for the 83 Aboriginal Australian mitogenomes reported in Malaspinas *et al*.^18^ were not available. Mitogenomes from other populations (non-Australians) were taken from Ingman, *et al*.^38^, Redd, *et al*.^22^, Ingman and Gyllensten^37^, Pierson, *et al*.^55^, Friedlaender, *et al*.^51^, Hudjashov, *et al*.^9^, Tabbada, *et al*.^31^, Delfin, *et al*.^32^ and Gomes, *et al*.^34^. A Maximum-Likelihood tree was constructed using MEGA v6.0^76^ with the GTR+I+G nucleotide model and 1000 bootstraps.

## Additional Information

**Accession codes:** GenBank, http://www.ncbi.nlm.gov/Genbank/ (for sequences [accession numbers KY595546-KY595672])

**How to cite this article:** Nagle, N. *et al*. Aboriginal Australian mitochondrial genome variation – an increased understanding of population antiquity and diversity. *Sci. Rep.* **7**, 43041; doi: 10.1038/srep43041 (2017).

**Publisher's note:** Springer Nature remains neutral with regard to jurisdictional claims in published maps and institutional affiliations.

## Supplementary Material

Supplementary Information

Supplementary Figure 1

Supplementary Figure 2

Supplementary Figure 3

## Figures and Tables

**Table 1:** TMRCAs of Aboriginal Australian mitochondrial haplogroups.

Present Study						Other Publications			
Haplogroup	n	Fu *et al*.^57^		Soares *et al*.^56^		Behar *et al*.^50^^a^	Hudjashov *et al*.^9^^b^	van Holst Pellekaan^30^^c,d^	
		Median (KY)	95% HPD (KY)	Median (KY)	95% CI (KY)	TMRCA ± SD (KY)	TMRCA ± SD (KY)	TMRCA ± SD (KY) – Coding region	TMRCA ± SD (KY) – Coding region
M15	2	31	19–43	67	46–89	—	—	—	—
M42a	15	36	27–46	39	31–47	—	41 ± 9	34 ± 6	46 ± 9
M42c	19	46	35–58	43	29–58	—	—	—	—
M42b	14	46	35–58	53	34–72	40 ± 7	—	—	—
M42a'c	34	53	42–66	50	39–62	—	—	—	—
Q	34	44	34–55	53	37–68	37 ± 6	32 ± 6	—	—
Q1	18	23	17–30	24	17–32	18 ± 7	21 ± 6	—	—
Q2	11	34	24–44	27	20–35	29 ± 7	30 ± 9	—	—
Q3	5	31	22–41	35	27–43	31 ± 6	21 ± 5	—	—
N13	4	32	22–44	38	26–52	—	—	—	—
O	9	37	25–50	43	28–59	52 ± 6	17 ± 7	—	—
O1	5	25	16–36	20	12–29	17 ± 8	—	—	—
O1a	4	12	7–18	19	11–29	—	—	—	—
O1a1	3	7	3–12	17	10–24	—	—	—	—
O2	4	24	14–35	22	13–32	—	—	—	—
S	48	51	40–64	49	39–59	53 ± 5	25 ± 5	40 ± 6	54 ± 8
S1	22	42	32–53	48	35–62	—	22 ± 7	—	—
S1a	8	22	15–29	20	12–29	—	—	32 ± 9	44 ± 12
S1b	13	21	15–28	29	22–37	—	—	—	—
S1b1	5	16	10–22	20	11–30	—	—	—	—
S1b2	5	10	5–16	9	2–17	—	—	—	—
S1b3	3	12	7–17	12	4–20	—	—	—	—
S2	21	32	24–42	34	25–44	39 ± 9	15 ± 5	—	—
S2a	14	25	18–34	29	20–38	—	—	—	—
S2b	7	26	18–35	30	19–42	—	—	—	—
S3	2	3	1–7	8	0–17	2 ± 2	—	—	—
P	112	60	50–73	62	54–70	55 ± 2	52 ± 6	—	—
P1	22	38	29–48	39	31–47	33 ± 6	30 ± 6	—	—
P1d	13	32	24–40	36	28–45	30 ± 6	—	—	—
P1e	8	26	16–37	30	17–44	—	—	—	—
P1e1	3	11	5–19	14	6–23	—	—	—	—
P1e1a	2	3	0.3–7	3	0–6	—	—	—	—
P2	8	39	28–50	29	20–38	—	13 ± 4	—	—
P2a	6	23	15–32	19	12–27	—	—	—	—
P2b	2	4	1–8	4	0–8	—	—	—	—
P3	10	43	33–54	44	26–62	41 ± 5	39 ± 8	—	—
P3a	4	26	17–35	6	0–13	25 ± 7	—	—	—
P3b	6	34	26–44	35	24–45	35 ± 6	—	—	—
P4	5	18	12–26	22	14–30	53 ± 4	66 ± 13	—	—
P4a	3	13	8–19	20	10–31	19 ± 6	26 ± 7	—	—
P5	24	31	23–41	28	18–39	—	—	—	—
P5a	21	23	16–30	22	13–30	—	—	—	—
P5a1	18	19	14–25	19	11–27	—	—	—	—
P5a2	3	17	10–24	20	11–30	—	—	—	—
P5b	3	19	12–28	21	10–32	—	—	—	—
P6	5	43	32–56	54	38–70	48 ± 7	—	—	—
P6a	2	12	5–19	16	7–26	—	—	—	—
P6b	3	32	22–43	44	26–63	—	—	—	—
P8	3	49	37–61	47	36–59	—	—	—	—
P8a	2	16	8–25	19	9–30	—	—	—	—
P9	10	40	30–52	59	38–82	—	—	—	—
P10	4	4	1–9	7	2–13	—	—	—	—
P11	8	50	39–62	39	29–48	—	—	—	—
P12	12	46	36–57	57	38–77	—	—	—	—
P12a	3	32	23–42	32	19–45	—	—	—	—
P12b	9	15	9–22	15	7–24	—	—	—	—

^a^One substitution in 3,624 years (Soares *et al*.^56^). ^b^One synonymous substitution in 6,764 years (Kivisild *et al*.^44^). ^c^One substitution in 3,810 years (Ingman and Gyllensten^37^). ^d^One substitution in 5,140 years (Mishmar *et al*., 2003).
