# Supplementary material for: Aboriginal Australian mitochondrial genome variation – an increased understanding of population antiquity and diversity
Source: Sci Rep. 2017 Mar 13;7:43041. doi: 10.1038/srep43041 (PMC5347126; doi:10.1038/srep43041)
Supplement: Supplementary Information [file srep43041-s1.pdf]

SUPPLEMENTARY INFORMATION

Title: Aboriginal Australian mitochondrial genome variation – an increased understanding of population antiquity and diversity

Authors:
Nano Nagle (1), Mannis van Oven (2), Stephen Wilcox (3), Sheila van Holst Pellekaan (4,5), Chris Tyler-Smith (6), Yali Xue (6), Kaye N. Ballantyne (2,7), Leah Wilcox (1), Luka Papac (1), Karen Cooke (1), Roland A.H van Oorschot (7), Peter McAllister (8), Lesley Williams (9), Manfred Kayser (2) Robert J. Mitchell (1)* and The Genographic Consortium (#)

Supplementary Table 1. Geographical distribution of Aboriginal Australian mitogenomes of the present study

	State											
Haplogroup	Victoria	%	New South Wales	%	Queensland	%	Tasmania	%	Western Australia	%	Total	%
Macrohaplogroup M												
M(xM42a)	2	33.33	1	7.14	17	16.50			1	50.00	21	16.54
M42a			1	7.14	10	9.71					11	8.66
Q					3	2.91					3	2.36
Macrohaplogroup N												
N*					2	1.94					2	1.57
O					4	3.88					4	3.15
S	1	16.67	6	42.86	24	23.30	1	50.00			32	25.20
Haplogroup R												
P	3	50.00	6	42.86	43	41.75	1	50.00	1	50.00	54	42.52
Total	6	4.72	14	11.02	103	81.10	2	1.57	2	1.57	127	

Supplementary Table 2. Distribution of mtDNA haplogroups in Aboriginal Australian populations after mtSNP typing

		State											
SNP Result	Haplogroup	Victoria	%	New South Wales	%	Queensland	%	Tasmania	%	Western Australia	%	Total	%
Macrohaplogroup M													
	M(xM42a)	5	33.33	9	18.37	45	14.52			3	75.00	62	16.27
	M42a	2	13.33	3	6.12	37	11.94					42	11.02
	Q			1	2.04	11	3.55					12	3.15
Macrohaplogroup N													
	N*					11	3.55					11	2.89
	O			4	8.16	8	2.58					12	3.15
	S	4	26.67	13	26.53	58	18.71	1	33.33	1	25.00	77	20.21
Haplogroup R													
	P	4	26.67	19	38.78	140	45.16	2	66.67			165	43.31
Total		15	3.94	49	12.86	310	81.36	3	0.79	4	1.05	381	

Supplementary Table 3. TMRCAs of Aboriginal Australian mitochondrial haplogroups and subhaplogroups

Haplogroup	Fu et al. (2013) Median (KY)	Fu et al. (2013) 95% HPD	Soares et al. (2009) Median (KY)	Soares et al. (2009) 95% CI (KY)
M	66	53 - 80	56	46 - 67
M15	31	19 - 43	67	46 - 89
M42a	36	27 - 46	39	31 - 47
M42a1	33	25 - 42	40	30 - 50
M42a1a	25	19 - 33	35	25 - 45
M42a1a1	16	8 - 24	19	8 - 30
M42a1a2	23	17 - 31	33	23 - 42
M42a1a2a	17	11 - 23	27	15 - 39
M42a1a2b	12	7 - 18	22	12 - 32
M42a1b	20	12 - 29	34	20 - 49
M42c	46	35 - 58	43	29 - 58
M42c1	7	2 - 14	7	1 - 13
M42c2	42	31 - 54	34	24 - 44
M42c2a	28	19 - 38	30	20 - 41
M42c2a1	14	8 - 20	17	9 - 25
M42c2a2	16	10 - 23	20	11 - 30
M42b	46	35 - 58	53	34 - 72
M42a'c	53	42 - 66	50	39 - 62
M42a'b'c	58	46 - 71	60	47 - 73
Q	44	34 - 55	53	37 - 68
Q1	23	17 - 30	24	17 - 32
Q2	34	24 - 44	27	20 - 35
Q3	31	22 - 41	35	27 - 43
N	67	54 - 81	66	53 - 80
N13	32	22 - 44	38	26 - 52
N13a	25	16 - 35	34	20 - 48
N13a1	11	5 - 18	15	6 - 24
O	37	25 - 50	43	28 - 59
O1	25	16 - 36	20	12 - 29
O1a	12	7 - 18	19	11 - 29
O1a1	7	3 - 12	17	10 - 24
O2	24	14 - 35	22	13 - 32
S	51	40 - 64	49	39 - 59
S1	42	32 - 53	48	35 - 62
S1a	22	15 - 29	20	12 - 29
S1a1	17	12 - 24	12	8 - 16
S1b	21	15 - 28	29	22 - 37
S1b1	16	10 - 22	20	11 - 30
S1b1a	10	6 - 16	15	6 - 24
S1b2	10	5 - 16	9	2 - 17
S1b3	12	7 - 17	12	4 - 20
S2	32	24 - 42	34	25 - 44
S2a	25	18 - 34	29	20 - 38
S2a1	18	12 - 25	21	12 - 31
S2a1a	11	7 - 16	12	6 - 19
S2a2	18	11 - 26	26	14 - 38
S2b	26	18 - 35	30	19 - 42
S2b1	16	9 - 25	18	10 - 27
S2b2	19	12 - 27	26	15 - 37
S3	3	1 - 7	8	0 - 17
P	60	50 - 73	62	54 - 70
P1	38	29 - 48	39	31 - 47
P1d	32	24 - 40	36	28 - 45
P1d1	23	16 - 31	33	21 - 45
P1d1b	19	12 - 26	30	17 - 43
P1d1b1	10	5 - 16	16	7 - 26
P1d2	26	19 - 35	28	15 - 41
P1e	26	16 - 37	30	17 - 44
P1e1	11	5 - 19	14	6 - 23
P1e1a	3	0.3 - 7	3	0 - 6
P2	39	28 - 50	29	20 - 38
P2a	23	15 - 32	19	12 - 27
P2b	4	1 - 8	4	0 - 8
P3	43	33 - 54	44	26 - 62
P3a	26	17 - 35	6	0 - 13
P3b	34	26 - 44	35	24 - 45
P3b1	30	22 - 39	30	20 - 41
P3b1a	20	13 - 28	11	0 - 22
P3b2	29	20 - 39	21	12 - 29
P4	18	12 - 26	22	14 - 30
P4a	13	8 - 19	20	10 - 31
P5	31	23 - 41	28	18 - 39
P5a	23	16 - 30	22	13 - 30
P5a1	19	14 - 25	19	11 - 27
P5a1a	15	11 - 20	14	8 - 20
P5a1a1	13	9 - 18	12	6 - 19
P5a2	17	10 - 24	20	11 - 30
P5b	19	12 - 28	21	10 - 32
P6	43	32 - 56	54	38 - 70
P6a	12	5 - 19	16	7 - 26
P6b	32	22 - 43	44	26 - 63
P8	49	37 - 61	47	36 - 59
P8a	16	8 - 25	19	9 - 30
P9	40	30 - 52	59	38 - 82
P9a	35	24 - 46	40	18 - 63
P10	4	1 - 9	7	2 - 13
P11	50	39 - 62	39	29 - 48
P11a	37	27 - 48	31	21 - 41
P11a1	18	11 - 26	20	13 - 28
P12	46	36 - 57	57	38 - 77
P12a	32	23 - 42	32	19 - 45
P12a1	24	16 - 32	16	9 - 24
P12b	15	9 - 22	15	7 - 24

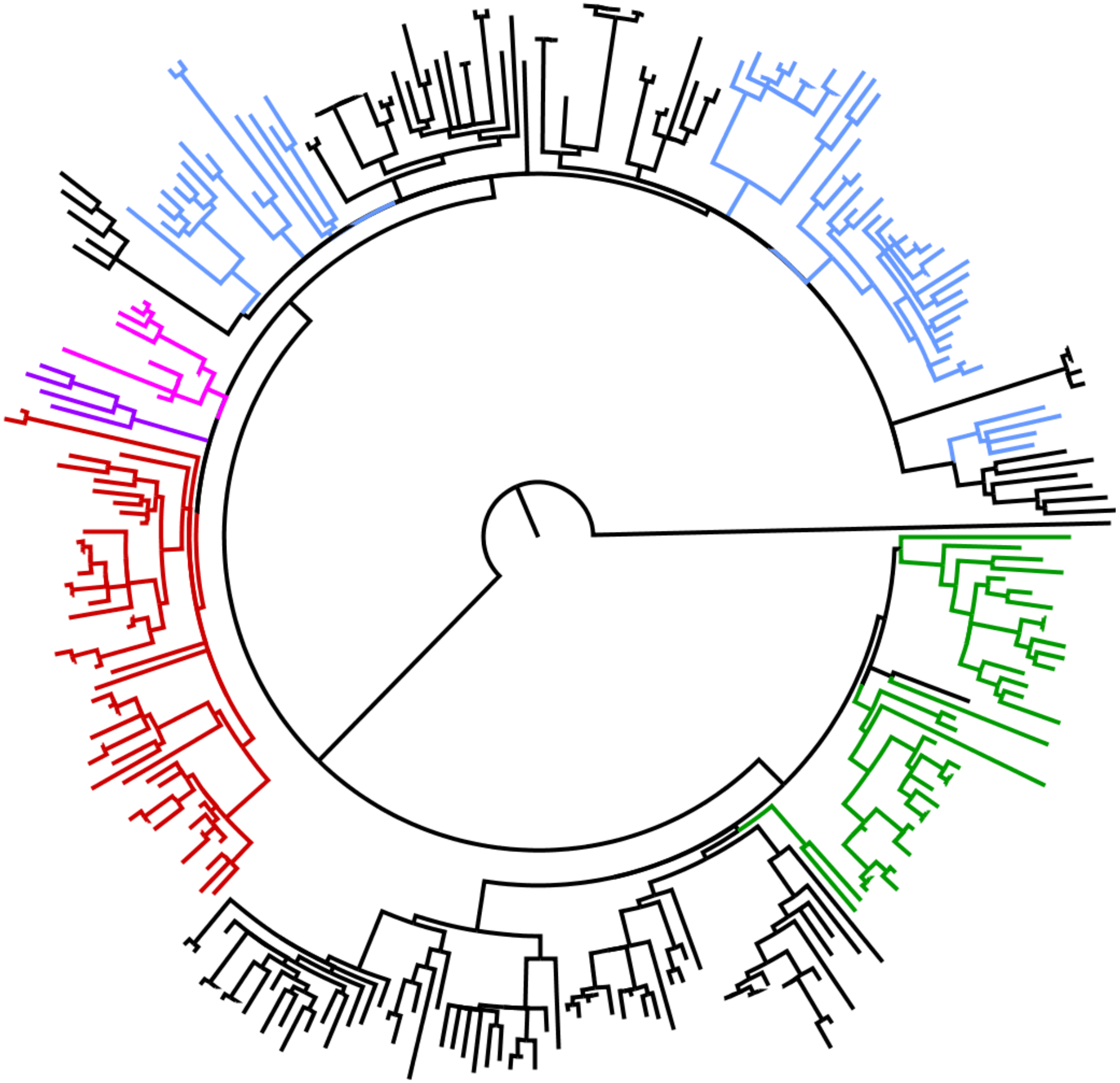

Supplementary Figure 4. A maximum likelihood phylogeny of Island Melanesian, Philippine, Indian and Aboriginal Australian mitochondrial haplogroups. Australian sequences are coloured as: M, green; S, red; O, magenta, N13, purple and P, light blue.

Supplementary Document: Sample Characteristics and Treatment

We cannot be certain that the mtDNAs reflect the ‘ancestral’ region where the samples were collected and this is especially true for mtDNA as traditionally females in many communities moved on marriage to their husband’s clan territory (the grandmother’s group affiliation could well differ from that of her granddaughter).

Subsequent to the European invasion there has been forced removal of Aboriginal Australians from their homelands into Government or religious settlements, restrictions on marriage and the forced removal of children from their parents. Added to this has been the progressive exodus from rural areas into the cities for employment. As a result of this turmoil much of the language and clan structure has dissolved, and this is especially so in the eastern States from which most of the samples are drawn. Therefore, seeking language or even tribal affiliation of people, most of whom have been separated from their traditional homeland and lifestyle from birth, would create a negative feeling among them. It is for this reason we report the variation only at State level as we feel confident that this is reasonably accurate. Any attempt at a reconstruction of the past genetic structure of Aboriginal Australia has to factor in this treatment for nearly 200 years.

Supplementary Experimental Procedures

1 Methods

1.1 Human mt DNA amplification

In order to create baits, the human mitogenome was broken into 12 overlapping PCR amplicon sets (see table below) using a subset of primer pairs from Rieder, et al. [1].

PCR name	Paper name	F primer start	R primer start	amplicon size
A	2F & 3R	1245	2669	1424
B	4F & 5R	2499	3961	1462
C	6F & 7R	3796	5420	1624
D	8F & 9R	5255	6642	1387
E	10F &11R	6469	8095	1626
F	12F & 13R	7937	9397	1460
G	14F & 15R	9230	10837	1607
H	16F &17R	10672	12076	1404
J	18F & 19R	11948	13507	1559
K	20F & 21R	13338	14998	1660
L	22F & 23R	14856	5	1720
M	24F & 1R	16240	1411	1742

Each PCR was undertaken using PCR conditions outlined in Rieder, et al. [1]

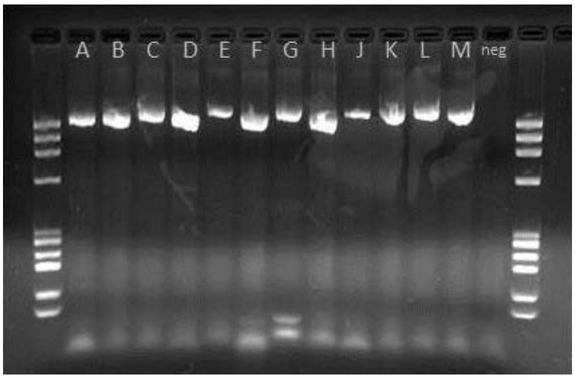

1.2 Preparing PCR amplicons for mitochondrial baits

To construct targeted mtDNA baits, the 12 PCR products were pooled in equal molar ratios and cleaned using AMPure XP beads (0.6x) to remove any DNA fragments below 500 bp. The cleaned pool was then sheared to a size range of 100-200 base pairs in size using the Covaris S220

instrument using the following process (peak power – 175, duty factor – 10%, cycles/burst – 200, 600 seconds). The sheared mtDNA was checked using a D1000 tape on the Agilent Tapestation 4000 instrument following manufacturer's instructions.

1.3 Clean up sheared DNA

The sheared mtDNA was then cleaned using Agencourt AMPure XP DNA beads according to manufacturer's instructions using 1.5x bead to sample concentration in order to remove small DNA fragments below 50 bp.

1.4 Preparing bait libraries

The human mtDNA bait libraries were prepared using the Illumina TruSeq Nano Library kit. The kit procedure was followed with the exception of when Illumina adapters were added during the ligation step, independently prepared T7 adapters were used instead. These T7 adapters were generated according to the protocol in Carpenter, et al. [2]. After the ligation and clean up step, the human cRNA mt baits were prepared according to the above paper.

1.5 Prepare human DNA libraries for capture

Mouthwash and Oragene DNA extracted samples were prepared using the same Illumina Truseq Nano Library kit, following the entire protocol. At the adapter ligation step, the adapters contained on a high output DNA adapter plate were used in order to increase sample multiplexing for next generation sequencing.

1.6 Capturing human mtDNA using human mt cRNA baits

The process of enriching human mtDNA for sequencing used the procedure from the aforementioned paper [2].

1.7 Next generation sequencing of enriched captured human mtDNA fragments

Once the captured mtDNA from each of the sample libraries was enriched, these captured DNA pools were used to seed the Illumina next generation sequencing protocol. The pool was quantified

using the Agilent Tapestation and the Qubit™ RNA assay kit for Qubit 2.0® Fluorometer (Life technologies). The indexed libraries were then prepared for paired end sequencing on a NextSeq instrument using the Mid Output 150 cycle kit (Illumina) as per manufacturer's instructions. The captured pools were sequenced using 2x 75 base reads from either end of the library insert.

1.8 Bioinformatics of the sequenced enriched captured human mtDNA fragments

Once the data was generated on the NextSeq instrument, the data was then demutiplexed to sort the data into the indexes used for each original mouthwash or Oragene sample. The files generated were fastq files for each index. These files were then used to align reads back to the human mt sequence from HG build 19. Once the alignment of the reads was finalized for each sample a report was generated to determine the coverage, depth and SNPs/SNVs that differed from the reference genome.

References:

1 Rieder, M. J., Taylor, S. L., Tobe, V. O. & Nickerson, D. A. Automating the identification of DNA variations using quality-based fluorescence re-sequencing: analysis of the human mitochondrial genome. *Nucleic Acids Research* **26**, 967-973 (1998).

2 Carpenter, M. L. *et al.* Pulling out the 1%: whole-genome capture for the targeted enrichment of ancient DNA sequencing libraries. *Am J Hum Genet* **93**, 852-864 (2013).
